# Graph embedding and unsupervised learning predict genomic sub-compartments from HiC chromatin interaction data

**DOI:** 10.1038/s41467-020-14974-x

**Published:** 2020-03-03

**Authors:** Haitham Ashoor, Xiaowen Chen, Wojciech Rosikiewicz, Jiahui Wang, Albert Cheng, Ping Wang, Yijun Ruan, Sheng Li

**Affiliations:** 1 The Jackson Laboratory for Genomic Medicine, Farmington, CT 06032 USA; 2 The Jackson Laboratory Cancer Center, Bar Harbor, ME 04609 USA; 3 Department of Genetics and Genome Sciences, University of Connecticut School of Medicine, Farmington, CT 06032 USA; 4 Department of Computer Science and Engineering, University of Connecticut, Storrs, CT 06269 USA

**Keywords:** Data processing, Machine learning, Software

## Abstract

Chromatin interaction studies can reveal how the genome is organized into spatially confined sub-compartments in the nucleus. However, accurately identifying sub-compartments from chromatin interaction data remains a challenge in computational biology. Here, we present Sub-Compartment Identifier (SCI), an algorithm that uses graph embedding followed by unsupervised learning to predict sub-compartments using Hi-C chromatin interaction data. We find that the network topological centrality and clustering performance of SCI sub-compartment predictions are superior to those of hidden Markov model (HMM) sub-compartment predictions. Moreover, using orthogonal Chromatin Interaction Analysis by in-situ Paired-End Tag Sequencing (ChIA-PET) data, we confirmed that SCI sub-compartment prediction outperforms HMM. We show that SCI-predicted sub-compartments have distinct epigenetic marks, transcriptional activities, and transcription factor enrichment. Moreover, we present a deep neural network to predict sub-compartments using epigenome, replication timing, and sequence data. Our neural network predicts more accurate sub-compartment predictions when SCI-determined sub-compartments are used as labels for training.

## Abstract (web summary)

Accurate identification of sub-compartments from chromatin interaction data remains a challenge. Here, the authors introduce an algorithm combining graph embedding and unsupervised learning to predict sub-compartments using Hi-C data.

## Introduction

The genome is hierarchically organized in three-dimensional (3D) space^1^, and chromosomal sequences can be mapped to one of two major compartments: compartment A is associated with open chromatin, and compartment B is associated with closed chromatin. The A/B compartments are predicted from chromatin interaction data, in particular, data generated by the genome-wide Hi-C chromatin interaction profiling technique, which combines proximity-based ligation with massively parallel sequencing^2^. Recent studies of additional data types, including methylation, DNase I hypersensitive sites, and single-cell ATAC-seq, have corroborated the existence of A/B compartments as functional units of genome organization^3^. The genome can be further subdivided into sub-compartments, where genomic regions within a given sub-compartment are more likely to interact with each other than with regions in different sub-compartments. Subsequent to the A/B compartment model, a three-sub-compartment model was introduced based on higher-resolution Hi-C inter-chromosomal interaction data^4^: one sub-compartment is a component of the open chromatin compartment; and the other two sub-compartments are components of closed chromatin compartment, one near centromeres and the other far from centromeres. The latest model proposes five sub-compartments based on the use of hidden Markov modeling (HMM) to cluster inter-chromosomal interactions^5^. This method identifies two active sub-compartments (A1 and A2) and three inactive sub-compartments (B1, B2, and B3) using deeply sequenced GM12878 cell line data. The model also shows that the five sub-compartments exhibit distinct epigenomic signatures. Since sub-compartments are essential for understanding higher-order 3D genome organization, and an increasing volume of genome-wide chromatin interaction data will soon be available through the ENCODE and 4D Nucleome consortia, there is a practical need for an easy-to-use, automated, and accurate sub-compartment predictor.

In addition to Hi-C, other types of genome-wide sequencing data have been used to predict the higher-order genome organization. For example, chromatin immunoprecipitation sequencing (ChIP-seq) data of 11 histone modifications and 73 transcription factors (TFs) have been used to predict sub-compartments with 63% test accuracy using a deep learning multi-class classifier^6^. Recently, TSA-Seq, a new genome-wide mapping method that estimates mean distances of chromosomal loci from nuclear structures, was used to predict several Mbp chromosome trajectories between nuclear structures^7^. These findings use sub-compartment assignments either for predictive model output labels or for evaluation of the distances between nuclear speckles. These studies require accurate sub-compartment determination. Also, a DNA methylation correlation matrix based on multiple replicates corroborates the A/B compartment model^3^. However, the potential of DNA methylation levels to predict nuclear sub-compartmentalization is still not clear.

Here, we introduce SCI, an algorithmic framework based on graph embedding^8^ and k-means clustering that predicts genomic sub-compartments from Hi-C data. We show that sub-compartment prediction by SCI is more accurate than other unsupervised algorithms. Furthermore, we use orthogonal data for external validation, including ChIA-PET chromatin interaction, transcriptome, and epigenome data. Lastly, using SCI output, we developed an epigenome-based (DNA methylation and histone modification) deep neural network model for sub-compartment classification.

## Results

### SCI overview

**Fig. 1:**
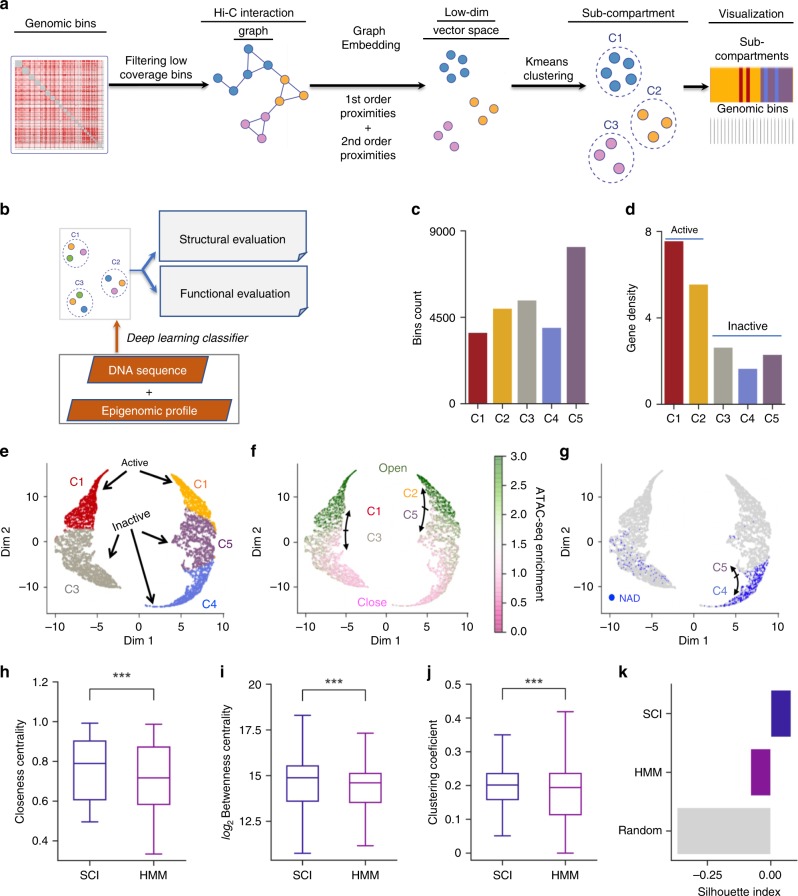
SCI utilizes a chromatin interaction graph to predict genomic sub-compartments. **a** The SCI workflow starts with a normalized Hi-C inter-chromosome matrix. From this matrix, the interaction graph is constructed; nodes with the same color represent bins from the same chromosome. After building the interaction graph, the graph embedding step transforms the graph into a lower-dimensional space. Finally, *k*-means clustering is used to cluster node representations. **b** SCI-predicted sub-compartments are validated using structural and functional properties and serve as class labels to train a sub-compartment classifier based on a feature matrix compiled with features of DNA sequence and epigenomic profiles (methylation and histone modifications), and replication timing data. **c** Bar plot of genomic bin count for each sub-compartment. **d** Bar plot of gene density measured as the number of genes per genomic bin for each sub-compartment. Sub-compartments associated with open chromatin (C1 and C2) show higher gene density compared to sub-compartments associated with closed chromatin (C3-C5). **e** UMAP 2-D projection of SCI’s embedding with 100 dimensions showing clustered sub-compartments for GM12878 Hi-C data. **f** ATAC-seq enrichment evaluating open and closed chromatin states across all five sub-compartments. **g** Distribution of nucleolar-associating domains (NADs) between C4 and C5. **h**–**k** Comparison of clustering quality metrics between SCI and HMM: (**h**) closeness centrality, (**i**) betweenness centrality, (**j**) clustering coefficient and (**k**) Silhouette index. For the boxplots, the top and bottom lines of each box represent the 75th and 25th percentiles of the samples, respectively. The line inside each box represents the median of the samples. The upper and lower lines above and below the boxes are the whiskers. Asterisks (***) represent *p*-values < 2.2e−16 using Mann–Whitney test.

To predict sub-compartments, SCI starts with a normalized, genome-wide Hi-C inter-chromosomal contact matrix and constructs a Hi-C interaction graph, where each node on the graph represents a genomic bin. If two bins are interacting, an edge connecting them is added to the graph, and the weight of the edge corresponds to the normalized Hi-C sequencing read count between the two bins. Then, SCI uses graph embedding^8^ to project the interaction graph into a lower-dimensional vector space for k-means clustering to predict sub-compartments (Fig. 1a).

Specifically, SCI adapts a graph-embedding method termed LINE^8^ to project an otherwise high-dimensional graph structure into a lower-dimensional space that describes the chromatin interactions. LINE utilizes first-order and second-order proximities of graph vertices to reduce dimensionality and promote efficient clustering.

The number of sub-compartments in the nucleus is jointly determined by (a) the spatial location of chromatin and (b) chromatin interactions. Importantly, local epigenetic status can impact chromatin interactions^9^. Using gap statistics to determine the optimal number of sub-compartments, we obtained nine as the optimal number of sub-compartments (named C1–C9). However, some of these sub-compartments are enriched for similar epigenetic modifications (C3 and C4; C5 and C6), suggesting that sub-compartments can be spatially separated and functionally comparable—they still show comparable levels of transcriptional potential (Supplementary Fig. 1). To allow for direct comparison of SCI with other computational methods for predicting sub-compartments, we chose to use 5 clusters in subsequent SCI performance evaluations. Importantly, SCI offers users the flexibility to select the number of sub-compartments based on either their preference or the optimal number calculated by gap statistics.

We then assessed the performance of SCI based on structural and functional evaluations (Fig. 1b). We also developed a deep neural network classifier for predicting sub-compartments using DNA sequencing data and epigenomic profiles (Fig. 1b).

### SCI outperforms alternative algorithms

We compared sub-compartments predicted by SCI to those predicted by HMM (reported in Rao et al. 2014) and by k-means clustering applied directly to an inter-chromosomal interaction matrix (Kmeans_Yaffe, reported in Yaffe, E. and Tanay, A., 2011). We tested each algorithm using data from Rao et al., who generated 4.9 billion Hi-C reads from GM12878, a human lymphoblastoid ENCODE cell line.

When given the same parameter (*k* = 5), SCI predicted comparable number of sub-compartments as that predicted by HMM (C1-C5; Fig. 1c, Supplementary Fig. 3); however, Kmeans_Yaffe failed to predict five sub-compartments, as it placed genomic bins in four sub-compartments instead of 5 (Supplementary Fig. 2). Following the convention of Rao et al., we reordered sub-compartments predicted by SCI such that sub-compartments C1 and C2 correspond to open chromatin sub-compartments reported by Rao et al. (A1 and A2), while sub-compartments C3, C4, and C5 correspond to closed chromatin sub-compartments reported by Rao et al. (B1, B2, and B3). Sub-compartments C1 and C2 have higher gene density compared to the other three sub-compartments (Fig. 1d), which is consistent with the previous reports^10^.

Our UMAP visualization of SCI embedding (Fig. 1e) shows: (i) a clear distinction between sub-compartments C1 (in red) and C2 (in yellow), which was indirectly reported in the literature by measuring the distance from lamina-associated domains (LADs)^11^; and (ii), clear separation of the inactive sub-compartment C3 (in gray) from the inactive sub-compartments C4 (in purple) and C5 (in blue). We further showed that the open chromatin-associated sub-compartments (C1 and C2) have higher enrichment of ATAC-seq compared to the inactive compartments (Fig. 1f, open-chromatin sub-compartment C1 compared to closed-chromatin sub-compartment C3, and open-chromatin sub-compartment C2 compared to closed-chromatin sub-compartment C5). Moreover, we show that C4 is more highly enriched for nucleolar-associating chromosomal domains (NADs)^12^ compared to C5 (Fig. 1g), indicating that C4 is located in the nucleolus while C5 is likely not. Thus, SCI-predicted sub-compartments exhibit distinct chromatin characteristics.

Genomic regions that map to the same sub-compartment based on the graph embedding of SCI are expected to be in proximity to each other in 3D space. To test this, we examined intra-sub-compartment, inter-chromosomal chromatin interaction network topology features, including network centrality (closeness and betweenness) and the clustering coefficient. Compared to the HMM approach, SCI showed significantly higher closeness centrality (*p*-values < 2.2e−16, Mann–Whitney test) and betweenness centrality, and a higher graph clustering coefficient within sub-compartments (Fig. 1h–j), with improvements between 8–10%. In addition, we found that open sub-compartments (C1 and C2) show higher centrality values for chromatin interactions compared to the closed sub-compartments (C3, C4, C5) (Supplementary Fig. 3), indicating that heterochromatin is more spatially constrained than euchromatin. These topological network features indicate higher chromatin interactions between genomic regions within the same SCI-predicted sub-compartments. To further evaluate the clustering performance of SCI and HMM, we calculated their Silhouette indices, which measure the consistency within clusters of data^13^. The Silhouette index ranges from −1 to 1, where a higher value indicates better clustering performance. SCI improved the Silhouette index over the previous HMM sub-compartment annotation by 8% (Fig. 1k). Moreover, we calculated the Davies-Bouldin index, where lower values are better, and SCI improved the Davies-Bouldin index by 7% (Supplementary Fig. 4). We also compared LINE to the state-of-the-art graph embedding algorithms HOPE^14^ and DeepWalk^15^. LINE-based cluster results had a better Silhouette index and centrality measures compared to the alternative graph embedding methods (Supplementary Fig. 5).

### SCI performance validated with ChIA-PET data

**Fig. 2:**
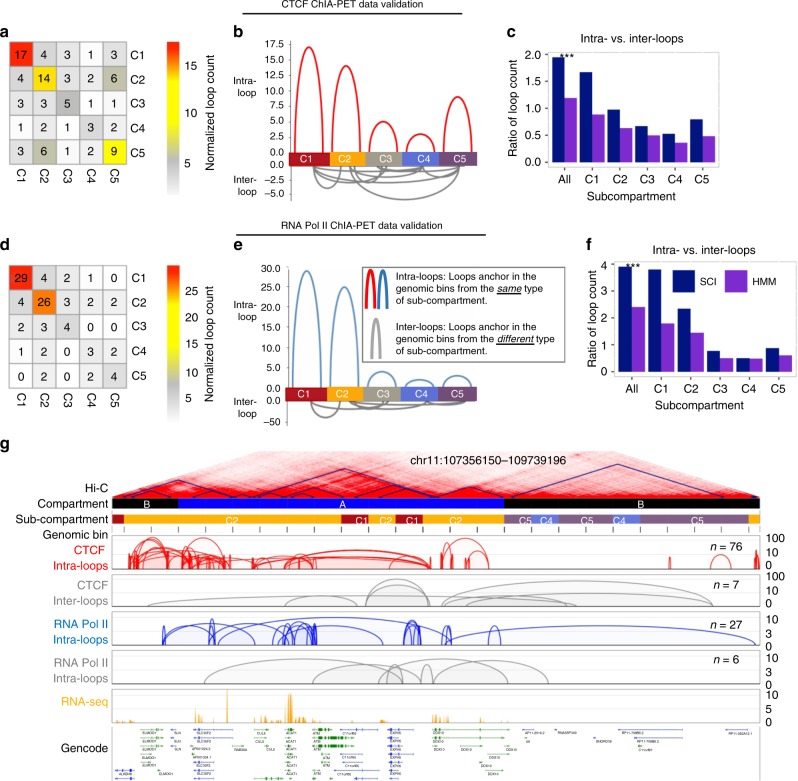
External validation of SCI-predicted sub-compartments using orthogonal ChIA-PET chromatin interaction data. Validation using CTCF ChIA-PET (**a**–**c**) and RNAPII ChIA-PET (**d**–**f**)**. a**, **d** Heatmaps of ChIA-PET loop counts per predicted sub-compartment, based on the mapping of loop anchors to genomic bins. Loop counts normalized genome-wide using sub-compartments determined by SCI. **b**, **e** Plots visualizing the abundance of ChIA-PET loops across SCI-predicted sub-compartments. **c**, **f** Bar plots comparing the ratios of ChIA-PET loops connecting genomic bins within the same sub-compartments to the loops connecting genomic bins from different sub-compartments, based on the sub-compartment predictions by SCI and HMM. **g** Example regions of SCI sub-compartment predictions that have more intra-sub-compartment loops (intra-loops) than inter-sub-compartment loops (inter-loops) supported by CTCF and RNAPII ChIA-PET. Asterisks (***) represent *p*-value < 2.2 × 10^−16^ using Fisher’s exact test.

To further validate that genomic regions from any given Hi-C-based SCI-predicted sub-compartment have higher chromatin interaction frequencies than those from different sub-compartments, we applied SCI to datasets from an orthogonal chromatin interaction assay, Chromatin Interaction Analysis by Paired-End Tag Sequencing (ChIA-PET). ChIA-PET measures chromatin interactions associated with specific protein factors^16^. We used two different ChIA-PET datasets: the first captures interactions associated with CCCTC-binding factor (CTCF), a chromatin-binding protein associated with more than 70% of the total chromatin interactions^17^; and the second captures interactions associated with RNA polymerase II (RNAPII), which coordinates communication between promoters and their distal regulatory elements. We then compared the chromatin interactions in GM12878 cells measured by CTCF and RNAPII ChIA-PET to quantify two types of chromatin interactions: (i) intra-loops, which are defined as ChIA-PET chromatin loops that connect two genomic bins that have the same sub-compartment predictions; and (ii) inter-loops, which are defined as ChIA-PET chromatin loops that connect two genomic bins that have different sub-compartment predictions (Fig. 2). We showed that there are more CTCF ChIA-PET intra-loops (the diagonal of the heatmap in Fig. 2a and the top panel of Fig. 2b and Supplementary Fig. 6) than CTCF ChIA-PET inter-loops. SCI-detected sub-compartments achieved a higher intra-loop vs. inter-loop ratio of normalized CTCF ChIA-PET loop counts compared to HMM-detected sub-compartments (Fig. 2c, *p*-value < 2.2 × 10^−16^, Fisher’s exact test). RNAPII ChIA-PET data corroborated a higher ratio of intra-loops vs. inter-loops using SCI compared to HMM (Fig. 2d–f). This equated to a 60% and 74% more robust performance for SCI compared to HMM for CTCF and RNAPII loop ratios, respectively. We then visualized the ChIA-PET loops in an example region with more intra-loops than inter-loops (Fig. 2g). Together, these data demonstrate that, compared to HMM-prediction of sub-compartments, SCI-prediction of sub-compartments achieves better clustering performance and tighter network topology structure.

### Functional assessment of sub-compartments

**Fig. 3:**
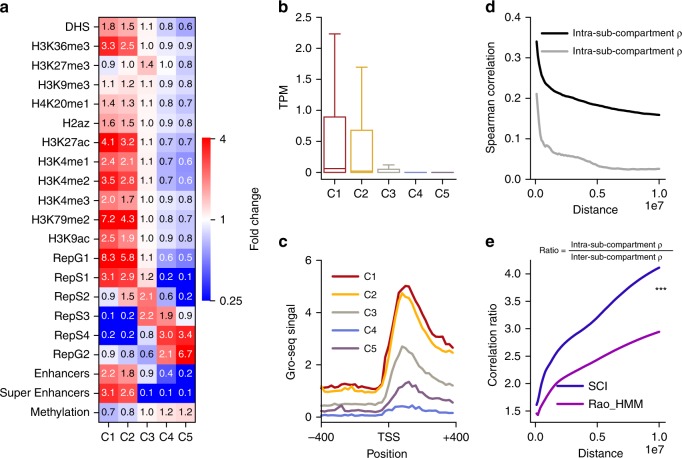
SCI sub-compartments have distinct genomic features. **a** Heatmap of distinct epigenomic sub-compartments predicted by SCI. **b** Boxplot of gene expression in transcripts per million (TPM) for different compartments. C1 and C2 have higher gene expression compared to other sub-compartments. **c** Cumulative GRO-seq signal over the promoter regions for all the genes in each sub-compartment. **d** Pairwise gene-expression Spearman’s correlations vs. distance between gene pairs within the same sub-compartment (in black) vs. gene pairs across sub-compartments (gray) within 10 Mbps. **e** Spearman’s correlation coefficient ratio (*y*-axis) of gene pairs within the same sub-compartment (in black) vs. gene pairs across sub-compartments plotted against genome distance (*x*-axis) for SCI (blue) and HMM prediction. Asterisks (***) represent *p*-value < 2.2e−16 using paired Wilcoxon test.

To compare the functional properties of sub-compartments predicted by SCI with those of sub-compartments predicted by other methods, we assessed enrichment for epigenomic marks and replication timing within sub-compartments. Data comprised DNase hypersensitive sites (DHS) (open chromatin), ten histone modifications, enhancer annotations, super-enhancer annotations, DNA methylation, and six replication timing datasets (Fig. 3a, Supplementary Fig. 7). In general, transcriptionally active sub-compartments are expected to have higher enrichment for DHS and the histone modifications (H3K27ac and H3K36me), compared to transcriptionally inactive sub-compartments. While inactive sub-compartments are expected to have higher enrichment for H3K27me3 and DNA methylation. We found that SCI-predicted sub-compartments satisfied these expectations (Fig. 3a). In contrast, HMM-predicted sub-compartments exhibited higher enrichment for DHS in C3 (inactive) than in C2 (active) (Supplementary Fig. 7). We observed higher enrichment for enhancers and super-enhancers in the C1 and C2 sub-compartments predicted by both SCI and HMM (Fig. 3a, Supplementary Fig. 7), consistent with transcriptionally active genomic regions.

Genes in close 3D spatial proximity to one another are more likely to have synchronized transcriptional control compared to those that are in different sub-compartments^18^. Therefore, we examined transcriptional activity in SCI-predicted sub-compartments. We assessed gene-expression levels using ENCODE RNA-seq data of GM12878 cells. Genes within C1 and C2 had significantly higher transcript per million (TPM) values (*p*-value < 0.01 based on ANOVA) compared to genes within C3-5, consistent with their epigenetic regulatory status (Fig. 3b). Furthermore, we evaluated the generation of nascent RNA using Global run-on sequencing (GRO-seq)^19^. The GRO-seq data confirmed that transcriptional activity in C1 and C2 is significantly higher (fold change > 3, *p*-value < 0.01 based on ANOVA) than that in sub-compartments C3-5 (Fig. 3c).

We hypothesize that the expression of genes within the same sub-compartment is more highly coordinated than that of genes across different sub-compartments. Therefore, we calculated pairwise Spearman’s correlation of gene expression for gene pairs within the same sub-compartment and across sub-compartments. We observed higher pairwise correlation values of gene expression for genes falling into bins within the same sub-compartment (intra-compartment correlation) compared to genes in different sub-compartments (inter-compartment correlation) (Fig. 3d). We also showed that SCI achieved a significantly higher (*P*-value < 2.2e−16, paired Wilcoxon test) intra-compartment correlation to the inter-compartment correlation ratio compared to HMM. As shown in Fig. 3e, SCI performs 40% better than HMM with respect to pairwise gene-expression correlation for functional evaluation of sub-compartments.

**Fig. 4:**
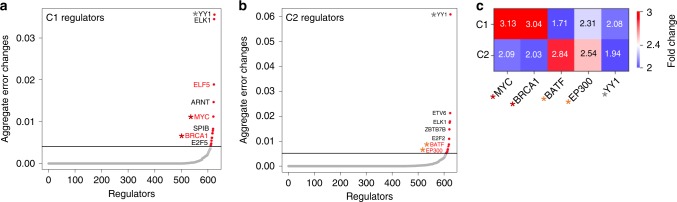
Sub-compartment-specific transcription factor prediction. **a**–**b** Scatter plot showing sub-compartment-specific transcription factors (TFs) participating in transcriptional regulation predicted by elastic net regression for C1 and C2 sub-compartment. The star indicates TFs with the ChIP-seq data available from ENCODE (red: C1-specific regulators, orange: C2-specific regulator, and gray: shared regulator). **c** Heatmap of ChIP-seq enrichment for MYC, BRCA1, BATF, EP300, and YY1 TFs across sub-compartments.

Folding the genome into sub-compartments may optimize the efficient usage of transcriptional regulatory elements. If true, we would expect to see sub-compartment-specific TF enrichment. To test this, we assessed TF enrichment using elastic net regression, which infers the regulatory elements that control transcription for each sub-compartment^20^. Based on feature dependency analysis^20^ (see Methods section for details), we identified sub-compartment-specific TFs that can be used to predict gene-expression levels in each sub-compartment (Supplementary Table 1). We also identified sub-compartment-specific TFs (red text in Fig. 4a, b). To further validate C1- and C2-specific TFs, we evaluated ENCODE ChIP-seq data that was available for MYC, EP300, and BRCA1 TFs. We found that ChIP-seq-based TF enrichment patterns agree with the predicted sub-compartment-specific TFs, where MYC and BRCA1 are enriched in C1, and BATF and EP300 are enriched in C2 (Fig. 4c). In addition, we observed that YY1, a TF common to both C1 and C2, is not enriched in either sub-compartment. These results indicate that SCI can identify chromatin hierarchical sub-compartments that parse into transcriptional regulatory units.

### Deep neural network for predicting sub-compartments

Recently, models have been proposed for predicting sub-compartment-level genome structure from the DNA sequence, ChIP-seq data^6^, and other 3D genome assays^7^. One approach uses a 450 K-based DNA methylation correlation matrix from multiple replicates (*n* ≥ 62) to identify genome-wide A/B compartments. However, there is no available model for sub-compartment structure prediction using a single DNA methylation library.

**Fig. 5:**
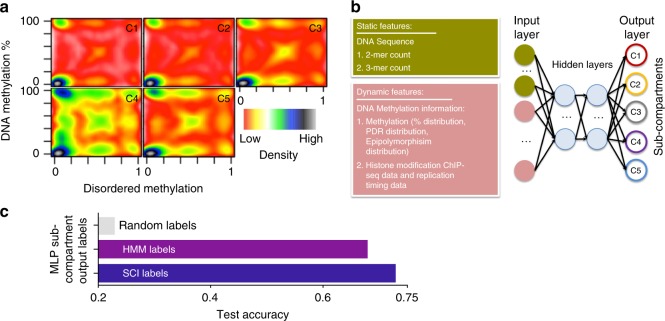
Deep neural network predicting sub-compartments using DNA methylation. **a** Smoothed scatter plot showing the distribution of the percentage of DNA methylation and the proportion of disordered DNA methylation reads for all sub-compartments. **b** The schematic diagram for the constructed a deep neural network (DNN) model to predict sub-compartments from methylation and DNA sequence features. **c** Bar plot showing the accuracy of sub-compartment prediction using random classifier trained with random labels, HMM sub-compartment annotation, or SCI sub-compartment annotation.

Using SCI predictions, the five sub-compartments showed distinct epigenome signatures (Fig. 3a), DNA methylation percentage distributions, and percentage of disordered reads, the latter of which is a measurement of epigenome stochasticity^21^ (Fig. 5a). Building on these distinctions, we developed an epigenome-based classifier to predict sub-compartments based on ChIP-seq epigenomic marks and whole-genome bisulfite sequencing data, using a deep neural network (DNN) (Fig. 5b). We combined dynamic epigenomic features and static DNA sequence features to design a deep neural network (DNN). We sliced the data set into a training set (80%) and test set (20%). The model accuracy reached higher test accuracy (0.73) using SCI-detected class labels than using HMM-detected class labels (0.68) or using randomized class labels (0.23) with the same class sizes (Fig. 5c). In addition, we developed a more compact model using only DNA methylation and DNA based features, which was able to predict genomic sub-compartments with 0.66 of accuracy. Because the model interpretability of DNN is limited, we used a gradient-boosted trees model (XGBoost) classifier and random forest machine learning methods to derive a feature importance score for our features. The accuracy of the XGBoost classifier is 0.69, and the accuracy of the random forest method is 0.67, each of which is slightly lower than the accuracy of our DNN model. The XGBoost and random forest methods agreed that histone modification features followed by DNA methylation distribution are the most important feature in the dataset (Supplementary Fig. 8).

Our sub-compartment DNN predictors outperformed recently developed model^6^ using 84 ChIP-seq libraries for histone modifications and TFs using the same training/test data split (training data is from odd-numbered chromosomes and test data is from even-numbered chromosomes) (Supplementary Table 2). Moreover, our reduced model using DNA methylation and DNA features outperformed their reduced model using 11 histone marks.

### Compartment prediction using Hi-C or ChIA-PET data

We also used SCI to predict A/B compartments from Hi-C and ChIA-PET data on two cell lines: GM128787 (human B-lymphocyte) and K562 (human lymphoblast). We predicted A/B compartments using a 100-kb resolution. We observed high agreement for A/B compartment prediction from both technologies (Supplementary Fig. 9) (agreement for GM12878 is 91% and agreement for K562 is 78%). We observed a high correlation (0.88 for GM12878 and 0.76 for K562) for the first eigenvector values between Hi-C and ChIA-PET. Moreover, we noted that enrichment for several open chromatin marks, histone marks, and replication timing data was similar using compartments called by Hi-C and ChIA-PET for both cell lines (Supplementary Fig. 10). We implemented the compartment predictor using static features (DNA sequence information) and dynamic features (DNA methylation information) as input for the deep neural network model and achieved 87% test accuracy (Supplementary Fig. 11).

## Discussion

Hi-C enables the elucidation of higher-order chromatin topology that suggests structural regulation of transcriptional control. Software exists for data pre-processing, chromatin loop calling, and topologically associating domain (TAD) predictions. However, there is no available software to examine compartment and sub-compartment structures. Furthermore, it has been shown that the TAD structure is rather stable among different cell types, while the structure of sub-compartments is highly variable among different cell types; this validates the need to study the cell type-specific functions of sub-compartments^22^. SCI identifies complex nuclear sub-compartments in a fully data-driven, unsupervised fashion.

In contrast to previous methods that infer sub-compartments using HMM, SCI more comprehensively utilizes network properties and thus may preserve the global structure of the chromatin interaction network. SCI formulates sub-compartment prediction as a graph-based problem and enables efficient utilization of Hi-C information via graph embedding. SCI considers both the first-order and second-order proximities, which complement the sparsity of first-order proximity in Hi-C data. We demonstrated that SCI outperforms other algorithms, such as HMM and Kmeans_Yaffe. We believe that the superior performance of SCI compared to other methods is due to its use of all the inter-chromosomal interactions, and efficient dimensionality reduction via graph embedding. Specifically, Rao_HMM uses only a subset of inter-chromosome interactions, and Kmeans_Yaffe does not perform a dimensionality reduction on the data. Importantly, we studied the functional relevance of sub-compartments as they relate to cellular processes. Moreover, we show TF enrichment in SCI-predicted sub-compartments.

Recently, various computational models have been proposed to predict genome organization from sequence and epigenomic data^6^ and other 3D genome assays^7^. We believe that SCI will further propel the development of such models by providing robust, accurate labels for genomic sub-compartments. As an example, we developed compartment and sub-compartment DNN predictors using static features from the DNA sequence of the reference genome and dynamic features from histone modifications and DNA methylation data and outperformed the published model^6^ using 84 ENCODE ChIP-seq libraries for histone modifications and TFs from the same cell line.

Importantly, the ENCODE and 4D Nucleome consortia are generating robust Hi-C datasets, and we anticipate that SCI will contribute to more efficient and accurate sub-compartment determination, and will improve our understanding of the complex interactions between DNA methylation, gene expression, and chromatin organization. Furthermore, tens of thousands of DNA methylation assays have been generated in large-scale studies such as The Cancer Genome Atlas, the Epigenome Roadmap, and Cancer Cell Line Encyclopedia. SCI will provide methods to maximize the value of these datasets and thereby enrich our understanding of the layers of gene-expression coordination that are important for development and disease states.

## Methods

### Background

All current sub-compartment prediction methods utilize an inter-chromosomal interaction matrix to predict sub-compartments. Two methods for prediction of genomic sub-compartments have been published. First model is the Yaffee and Tanay sub-compartment model (Yaffee_Kmeans). This model applies K-means clustering directly on a HiC interaction matrix. It modifies the distance calculation method such that it ignores all distance computation that involves intra-chromosomes entries. This model identifies three sub-compartments: one active and two inactive sub-compartments. The second model is Rao sub-compartments (HMM). The Rao sub-compartment prediction method focuses on one sub-set of inter-chromosomal interactions: the interactions between odd-numbered and even-numbered chromosomes. This method uses HMM to cluster the interactions between odd-numbered and even-numbered chromosomes in different runs. After performing cluster annotation for odd-numbered and even-numbered chromosomes, the Rao method combines those annotations into a single final cluster annotation based on enriched HiC interactions between the clusters of odd-numbered and even-numbered chromosomes.

### Graph-embedding methods

Given an undirected graph *G* = [*V*, *E*], where *V* represents genomic bins, and *E* represents Hi-C interactions, each edge (e) between *v*_*i*_ and *v*_*j*_ has weight *w*_*ij*_, which represents the normalized Hi-C read count. Graph-embedding approaches project graph G into a low dimension space Rd by calculating embedding matrix U (please refer to the following reviews for more detailed information about graph embedding^23–25^). We compared SCI graph embedding method to DeepWalk and HOPE graph embedding methods.

The Deepwalk method relies on performing random walks across the graph that have a specific length. These walks resemble node sequences in the graph, and this sequence is fed to the Word2Vec approach to derive the embedding of the graph vertices based on their context. The original DeepWalk implementation handles only unweighted graphs. We used a modified version of the algorithm to handle weighted graphs from (https://github.com/dongguosheng/deepwalk).

The HOPE method derives the graph embedding matrix ***U***, which minimizes the following objective function:

$$\left\| {S - u_su_t^T} \right\|_F^2$$

where ***S*** is a similarity matrix, and ***U*** is an embedding matrix where ***U*** = [***Us***, ***U****t*]. The similarity matrix can be defined using different similarity measures, including the Katz index and rooted page rank. For HOPE, we used the implementation of these algorithms from the GEM graph embedding package (https://github.com/palash1992/GEM). We used Katz index similarity measures in the original publication^14^.

### LINE and joint optimization

In order to project graph *G* into lower dimension *R*^*d*^, LINE defines two properties to preserve in the graph: first-order proximity and second-order proximity.

First-order proximity is defined as the pairwise proximity through edges between the vertices in graph *G*. LINE models first-order proximity between two vertices in graph *v*_*i*_ and *v*_*j*_:

1 $$p_1 = \frac{1}{{1 + e^{ - u_i^T \cdot u_j}}}$$

where *u*_*i*_ and *u*_*j*_ are vertices embedding into low-dimensional space *R*^*d*^. We define empirical edge distribution over the space *V* × *V*, $\hat p_1 = \frac{{w_{ij}}}{W}$ where $W = \mathop {\sum }\nolimits_{(i,j) \in E} w_{ij}$. To obtain the first-order embedding, LINE optimizes the KL divergence (omitting constants) between *p*_1_ and $\hat p_1$ as

2 $$O_1\,=\,- \mathop {\sum }\limits_{\left( {i,j} \right) \in E} w_{ij}{\mathrm{log}}(p_1(v_i,v_j))$$

For second-order proximity, the main assumption is that vertices connected to other vertices are similar. To calculate embedding based on the second-order proximity, LINE introduces the context concept. Each vertex in the graph is considered as a simple vertex or as a context to other vertices. LINE introduces two vectors u_i_ and ${\mathrm{u}}_{\mathrm{i}}^\prime$, where u_i_ is the representation for *v*_*i*_ as a vertex while ${\mathrm{u}}_{\mathrm{i}}^\prime$ is the representation for *v*_*i*_ as a context.

For each directed edge in the graph (*i*, *j*) (undirected edges are treated as two directed edges with opposite directions), LINE defines the probability of context *v*_*j*_ generated by vertex *v*_*i*_ as:

3 $$p_2\left( {v_j|v_i} \right) = \frac{{\exp \left( {{u^\prime}_j^T \cdot u_i} \right)}}{{\mathop {\sum }\nolimits_{k \in V} \exp \left( {{u^\prime}_k^T \cdot u_i} \right)}}$$

Empirical distribution $\hat p_2$ is defined as $\hat p_2 = \frac{{w_{ij}}}{{d_i}}$, where *d*i is the out-degree of node i.

LINE optimizes scaled (scaled by node degree) versions of the KL divergence between *p*_2_ and $\hat p_2$. It defines

4 $$O_2 = - \mathop {\sum }\limits_{w_{ij} \in E} w_{ij}\log \left( {p_2\left( {v_j{\mathrm{|}}v_i} \right)} \right)$$

SCI implements two approaches to combine defined objective functions *O*_1_ and *O*_2_. In the first approach, SCI optimizes *O*_1_ and *O*_2_ independently and then combines representation from both orders into a final representation. In the second approach (joint optimization), SCI combines first-order and second-order proximity objective functions into one function to define *O*_3_ as:

5 $$O_3 = \left( {1 - \alpha } \right)O_1 + \alpha O_2,\alpha \in \left[ {0,1} \right]$$

where *α* is a mixing parameter to determine the weight of first-order and second-order optimization functions.

It then optimizes both orders at the same time. An asynchronous gradient descent algorithm coupled with negative sampling is used to optimize all objective functions. The joint optimization showed a 30% increase in time efficiency (from 26 min to 18 min using GM12878 Hi-C data) and a slightly lower clustering performance of the joint optimization compared to separate optimization (measured by Silhouette index, Davies-Bouldin index, and centrality measures, Supplementary Fig. 12). Both separate and joint optimization are available as options in the SCI code.

### Evaluation for clustering methods

For each genomic bin we define Silhouette index (*s*_*i*_) as:

6 $$s_i = \frac{{b_i - a_i}}{{\max \left( {b_i,a_i} \right)}}$$

where *b*_*i*_ is the lowest average distance for genomic bin *i* for all other clusters, and *a*_*i*_ is the average distance from genomic bin *i* from all points in the same cluster.

We used the silhouette_score function from scikit-learn package version 0.19.2. We used Euclidean distance as our distance measure.

The Davies–Bouldin index (DBI) is defined as:

7 $${\mathrm{DBI}}\,=\,\frac{1}{k}\mathop {\sum }\limits_{i = 1}^k {\mathrm{max}}_{i \ne j}D_{ij}$$

where

8 $$D_{ij} = \frac{{d_i + d_j}}{{d_{ij}}}$$

Following the definition in ref. ^26^, we define *d*_*i*_ and *d*_*j*_ as the average distance between all pairs in clusters *i* and *j*. *d*_*ij*_ is the distance between all pairs between clusters *i* and *j*.

The clustering coefficient is defined as the ratio of the number of closed triplets (triangles) the node has to the number of all triplets that the node has. To adjust to the weighted graph, the weighted average for the closed triples is calculated^27^.

Closeness network centrality of a node is defined as the average length of the shortest path between the node and all other nodes in the graph. Closeness centrality for a node *u* in a graph with *n* nodes can be calculated as:

9 $$C\left( u \right) = \frac{{n - 1}}{{\mathop {\sum }\nolimits_{v = 1}^{n - 1} d(v,u)}}$$

where *d*(*v, u*) is the shortest path between nodes *u* and *v*.

Betweenness network centrality is defined as the number of times a node acts as a bridge along the shortest path between two other nodes. Betweenness centrality for a node u in a graph with *n* nodes can be calculated as:

10 $$C_B = \mathop {\sum }\limits_{s,t \in V} \frac{{\sigma (s,t|u)}}{{\sigma (s,t)}}$$

where *V* is the total number of nodes in the graph. $\sigma \left( {s,t} \right)$ is the number of shortest paths between nodes *s* and *t*. $\sigma (s,t|u)$ is the number of shortest paths between *s* and *t* passing through *u*.

Closeness and betweenness centralities were calculated using NetworkX version 2.1.

GM12878 CTCF and RNAPII ChIA-PET intra-chromosome loops supported by at least five paired-end reads were used for loop ratio calculation. A loop anchor is associated with a specific sub-compartment if it has an overlapping ratio greater than 50%. Next, we create sub-compartment loop interaction matrix ***M*** (*m*_*ij*_), where *m*_*ij*_ represents the number interaction between sub-compartments *i* and *j* detected using ChIA-PET data, such that the ChIA-PET loop left anchor falls in sub-compartment i and the right anchor falls in sub-compartment *j*. As there is no directionality difference between the left and right anchors, we create a symmetric compartment matrix ***M***_sym_ as the sum of ***M*** and ***M***^T^. Finally, a normalized matrix (***M***_norm_) is obtained by dividing ***M***_sym_ by the sum of its all elements.

An intra-sub-compartment loop was defined as a ChIA-PET loop with both anchors falling in genomic bins belonging to the same sub-compartment. Inter-sub-compartment loops were defined if two anchors of the loops fall in genomic bins from different sub-compartments. The ratio of the number of intra-sub-compartment and the number of inter-sub-compartment were then used to assess the connectivity of the predicted sub-compartments.

### Sub-compartment and compartment prediction using epigenome data

We developed machine learning and deep learning models for sub-compartment and compartment predictions using DNA sequence static features and epigenomic features. We utilized the XGBoost^28^ and random forest^29^ machine learning approaches to assess feature importance.

To predict sub-compartments from methylation and sequence-based data, we defined two types of features: static sequence-based features, and epigenome-based features. The static features include counting DNA-based 2-mers and 3-mers in each sub-compartment bin. The DNA methylation features include DNA methylation percentage distribution in each genomic bin, and epigenome instability or disordered DNA methylation patterns measured by the percentage of disordered reads (PDR) and epi-polymorphism. Also, we include the median signal for histone modification and replication timing data per genomic bin as features. Histone modifications data include H3K27ac, H3K27me3, H3K4me1, H3K4me2, H3K4me3, H3K79me2, H3K9ac, H3K9me3, H4K20me1, H3K36me3, and H2az marks. Replication timing data include: RepG1, RepG2, RepS1, RepS2, RepS3, and RepS4^30^. The model was constructed using the Keras framework (v2.2.4) and TensorFlow backend (v1.11.0).

### LINE hyperparameter for sub-compartment identification

We experimented with several hyperparameter settings for LINE. We performed hyperparameter selection over embedding size (size), number of negative samples (negative), and number of edges to be sampled to construct embedding (samples). We used the grid search to obtain the best parameters; below are the values used for each hyperparameter, with the selected values indicated in bold:

i. Size: **100**, 128, 200, 256, and 512
ii. Negative: 1, 2, 3, 4, **5**, 6, and 7
iii. Samples: 15, 20, **25**, 30, 40, 50

### Neural network hyper-parameter selection for sub-compartment and compartment prediction

We fine-tuned network hyper-parameters using a random search. Below is the list of hyper-parameters with their experimental values, with the selected values indicated in bold:

i. First layer number of nodes: [128, **256**, 512, 1024]
ii. Second layer number of nodes [**64**, 265, 512]
iii. Learning rate [0.01, **0.02**, 0.03, 0.04, 0.05]
iv. Second hidden layer drop-out rate [0.1, 0.2, 0.3, 0.4, 0.5, **0.60**, 0.7, 0.8, 0.9]
v. Optimizer: [SGD, RMSProp, **Adam**]
vi. Epochs: [**100**, 200, 300]
vii. Batch size: [32, 64, **128**, 265]

### Random forest and XGBoost hyper-parameter selection

We used the XGBoost Python library to build our XGBoost classifier. We used the grid search to fine-tune the number of boosting trees, and we experimented with the number of trees in the range of [20, 200] with a step of 10. We selected 100 trees based on cross-validation results using training data.

We used scikit-learn (scikit-learn.org) implementation for the random forest. We used the grid search to fine-tune the number of forest trees, and we experimented with the number of trees in the range of [20, 200] with a step of 10. We selected 130 trees based on cross-validation results using training data.

### Training and testing data design

Genomic bins (the data points) were split: 80% for training and 20% for testing. We applied random forest, XGBoost, and DNN models for sub-compartment and compartment classifications. We report accuracy as the performance for those models. For comparison with^6^ in Supplementary Table 2, we trained on odd-numbered chromosomes and tested on even-numbered chromosomes.

### Sub-compartment regulator inference

We obtained the DNA binding motifs of transcription factors (TFs) from the CIS-BP database^31^. We mapped TFs to the hg19 sequence using FIMO^32^, and we retained TF hits with FDR < 0.1. Then, we associated TF binding sites (TFBS) with genes if the predicted TFBS were within 2 kb of a gene TSS. We further filtered TFBS by selecting only those in accessible chromatin regions, as determined by ATAC-seq data. Finally, we constructed a features matrix where the genes represent samples, and all mapped TFs represent features, and where the count of TF hits associated with a given gene represents the feature value. We modified the RegulatorInference tool (https://bitbucket.org/leslielab/regulatorinference) to implement a sample-by-sample elastic net regression model to predict gene expression for each replicate using regulatory elements in gene promoters. We optimized the elastic net mixing parameter (alpha) using ten folds cross-validation. Based on the minimum squared error (mse), we picked an alpha value of 0.3 (Supplementary Fig. 13).

As a result of elastic net regression, we obtained a coefficient vector for TFs that represents the importance of the corresponding TF for the prediction of gene expression, while the sign of the coefficient can be interpreted as the predicted direction of regulation. We performed elastic net regression tasks separately for each sub-compartment.

Finally, we performed feature dependency analysis across samples to determine regulators that significantly account for compartment-specific gene expression in the regression models. In this process, the aggregate error change of one TF is calculated^20^. In summary, the aggregate error corresponds to the difference between the total regression error and the error contributed by a specific TF across the six different RNA-seq datasets used. To calculate the aggregate error for each elastic net model, initially, the entire error is measured using all learned TF coefficients. Then the TF coefficients are set to zero one at a time, then the error contributed by a specific TF is calculated. Lastly, the difference between both errors is calculated for each model and aggregated over the different models to define the final aggregate error.

The higher the aggregate error value, the most important the TF. If the aggregate error change of a TF is higher than the cutoff, it is considered an important TF. We used the default RegulatorInference tool cutoff, which corresponds to the mean of aggregate error changes across all TFs plus 1.5 times the standard deviation of aggregate error changes across all TFs.

### Epigenomic signal enrichment

The enrichment of the epigenome signal was calculated based on the description of (Rao et al., 2014). Briefly, to calculate enrichment for epigenomic data, we used bwtool^33^ to calculate the mean of the normalized signal for each histone mark in each bin downloaded from the ENCODE consortium data portal (see data availability). Then we computed enrichment as the ratio of the median of ChIP-seq signal for all of the bins in each sub-compartment divided by median of ChIP-seq signal of all of the bins genome-wide.

### Transcription factors enrichment

We obtained all ENCODE uniformly processed peaks from (http://hgdownload.cse.ucsc.edu/goldenPath/hg19/encodeDCC/wgEncodeAwgTfbsUniform/). We assigned peaks to different sub-compartments using bedtools^34^. We calculated peak enrichment in specific sub-compartments as the average peak abundance in a specific sub-compartment divided by genome-wide peak abundance. We evaluated the significance of the presence of a specific TF in a sub-compartment using the Mann-Whitney test compared to the genome-wide background. We considered only those TFs with corrected *p*-value <0.05 in at least one compartment. To evaluate the significance between C1 and C2 sub-compartments, we used a two-tailed Mann–Whitney test.

### Pairwise correlation among genomic bins using RNA-seq

Correlation of gene-expression levels within the same or different compartments was computed only for those genes expressed in at least one of the studied replicates. Each gene was represented as one TSS, corresponding with the 5’ end of the “gene” feature from Gencode V19 reference annotation^35^. All genes within a given maximum distance (e.g., 100 kb were joined into pairs. Spearman’s correlation coefficient was computed for each pair. Next, gene pairs were categorized as either intra-compartment, if both genes were located in the same type of compartment (e.g., C1), or as inter-compartment, if representative gene coordinates of a pair were located in different compartments. Finally, the average value of Spearman’s correlation coefficients was computed for all intra-compartment and inter-compartment pairs, considering only those for which results were statistically significant (i.e., *p*-value < 0.05). All of the above-described steps were conducted for the maximum range between pairs up to 10 Mb with a 100 Kb step.

### Gene-expression levels comparison using RNA-seq

We used transcript-per-million RNA-seq transcript quantification at the gene level in the box of Fig. 3b. We assigned gene TSSs to different sub-compartments, and then we plotted a boxplot using seaborn package version 0.9.0.

### Transcription efficiency measured by GRO-seq

We used bwtools to extract an aggregation plot^36^ for GRO-seq data around each TSS for each sub-compartment separately.

### DNA methylation data preprocessing

Briefly, whole genome bisulfite sequencing (WGBS) data were first trimmed of adapters using Trim-galore (version 0.4.0) [http://www.bioinformatics.babraham.ac.uk/projects/trim_galore/]. The base quality of the trimmed reads was checked with FastQC (version 0.11.5) [http://www.bioinformatics.babraham.ac.uk/projects/fastqc/]. The preprocessed reads were then aligned to human genome reference hg19 using Bismark (version 0.16.1)^37^. The aligned reads were then deduplicated, and the methylation call at each CpG site was determined by running the appropriate Bismark scripts. DNA methylation patterns were calculated using methclone^38^; epipolymorphism^39^ and PDR^21^ were calculated using epihet (https://www.bioconductor.org/packages/devel/bioc/html/epihet.html).

### ATAC-seq data processing

We used HMCan^40^ to obtain signal tracks from the alignment data.

### Enhancer and super-enhancers enrichment

We calculated enhancer and super-enhancer enrichment as the ratio between the expected value of observing a super-enhancer (or enhancer) per compartment and the expected value of observing a super enhancer (or enhancer) genome-wide.

### Reporting summary

Further information on research design is available in the Nature Research Reporting Summary linked to this article.

## Supplementary information

Supplementary Information

Reporting Summary

## Data Availability

We obtained processed (.hic) formatted Hi-C data for both GM12878 and K562 cell-lines from^5^ with GEO entry GSE63525. GM12878 ChIA-PET data accession code of 4D Nucleome consortium (https://commonfund.nih.gov/4dnucleome) is 4DNES7IB5LY9 (CTCF) and 4DNESZ25MOZV (RNAPII). We obtained raw ChIA-PET for CTCF and RNAPII factor for K562 cell-line from ENCODE. We processed raw files to produce loops follow the Links: https://www.encodeproject.org/experiments/ENCSR000BZY/ and https://www.encodeproject.org/experiments/ENCSR000CAC/. We used ATAC-seq data generated by^41^ GEO entry GSE47753. We downloaded signal bigwig tracks for DHS and histone modifications from the ENCODE consortium using the following link: http://ftp.ebi.ac.uk/pub/databases/ensembl/encode/integration_data_jan2011/byDataType/signal/jan2011/bigwig/. We downloaded super-enhancer annotations for GM12878 dbSuper database^42^ (https://asntech.org/dbsuper/). We obtained enhancer data for GM12878 from DENdb enhancers database^43^ (https://www.cbrc.kaust.edu.sa/dendb/), and used only high quality enhancers with a minimum score of 3 as assessed by DHS signal. We downloaded RNA-seq quantified gene expression files from ENCODE with the following GEO accessions: GSE88583, GSE88627, GSM958730, GSE90222, GSE78553, GSE78555. We downloaded GRO-seq signal tracks described in ref. ^19^ from GEO accession number GSE60456. For replication time data, we downloaded signal tracks from the ENCODE consortium using the following link: http://hgdownload.cse.ucsc.edu/goldenPath/hg19/encodeDCC/wgEncodeUwRepliSeq/. We obtained NAD enriched regions published in^12^ for hg18 reference. We used liftOver tool to map regions to hg19 reference. We only considered mapped regions with 0.95 confidence.

## Code availability

SCI code is available on https://github.com/TheJacksonLaboratory/sci and https://github.com/TheJacksonLaboratory/sci-DNN.
