# Supplementary material for: Graph embedding and unsupervised learning predict genomic sub-compartments from HiC chromatin interaction data
Source: Nat Commun. 2020 Mar 3;11:1173. doi: 10.1038/s41467-020-14974-x (PMC7054322; doi:10.1038/s41467-020-14974-x)
Supplement: Supplementary file 1 — Supplementary Information [file 41467_2020_14974_MOESM1_ESM.pdf]

Supplementary Information

Graph embedding and unsupervised learning predict genomic sub-compartments from HiC chromatin interaction data

Haitham Ashoor[1], Xiaowen Chen[1], Wojciech Rosikiewicz[1], Jiahui Wang[1], Albert Cheng[1], Ping Wang[1], Yijun Ruan[1,2,3], Sheng Li[1,2,3,4]

1. The Jackson Laboratory for Genomic Medicine, Farmington, CT, USA.
2. The Jackson Laboratory Cancer Center, Bar Harbor, ME, USA.
3. Department of Genetics and Genome Sciences, University of Connecticut School of Medicine, Farmington, CT, USA.
4. Department of Computer Science and Engineering, University of Connecticut, Storrs, Connecticut, CT, USA.

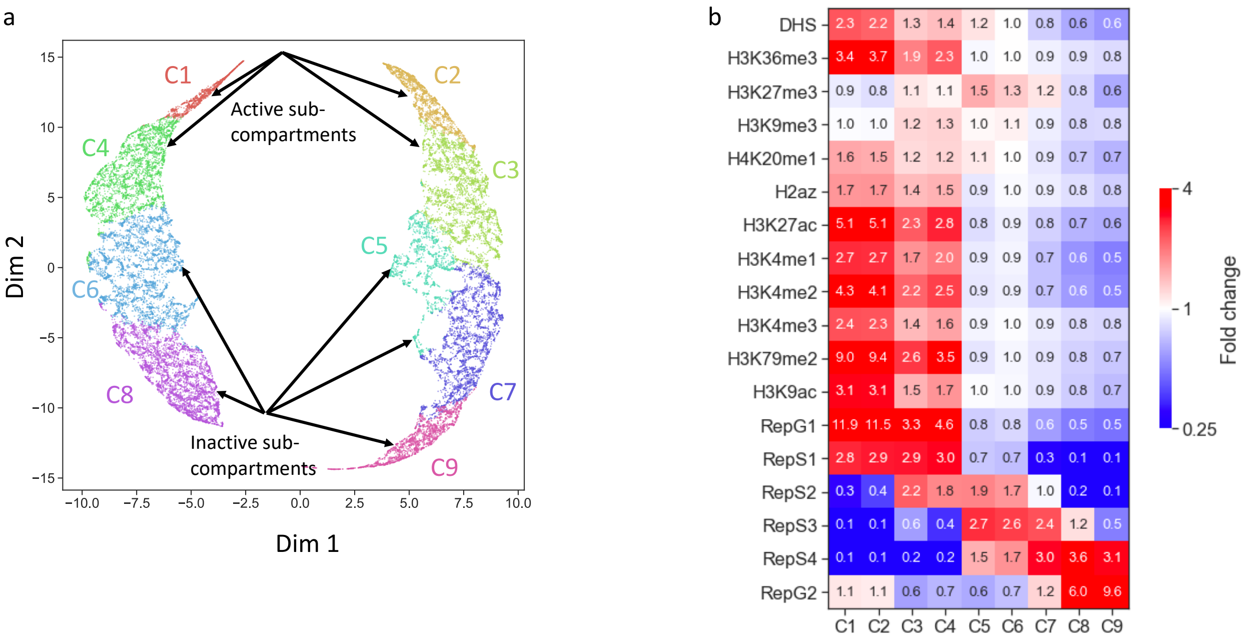

Supplementary Figure 1: SCI's nine sub-compartment results using gap statistics to determine the optimal number of sub-compartments. a) UMAP projection with nine sub-compartment annotations. **b)** Heatmap of epigenomic marks and replication timing enrichment across nine different sub-compartments.

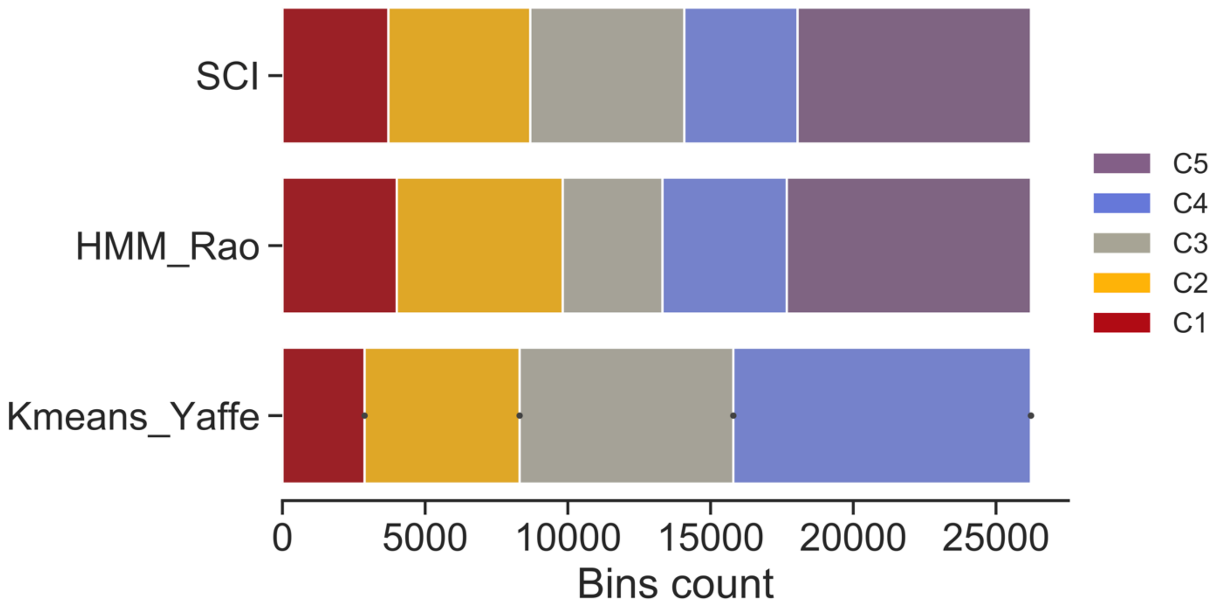

Supplementary Figure 2. Bar plot of proportions of genomic bins along sub-compartments for SCI, HMM_Rao, and Kmeans_Yaffe.

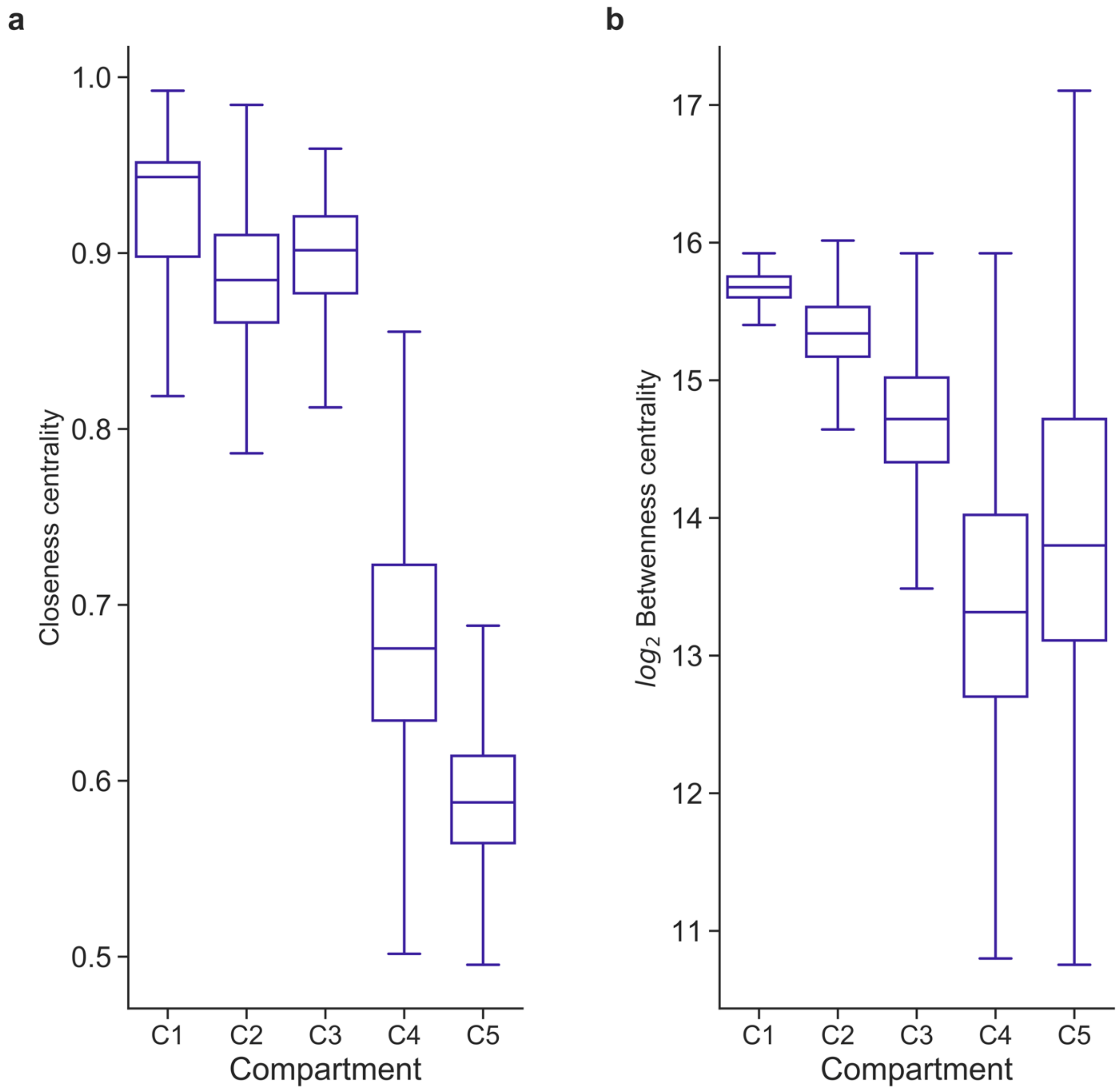

Supplementary Figure 3. Boxplot of the distribution of a) closeness centrality and **b)** betweenness centrality for SCI across sub-compartments. For the boxplots, the top and bottom lines of each box represent the 75th and 25th percentiles of the samples, respectively. The line inside each box represents the median of the samples. The upper and lower lines above and below the boxes are the whiskers.

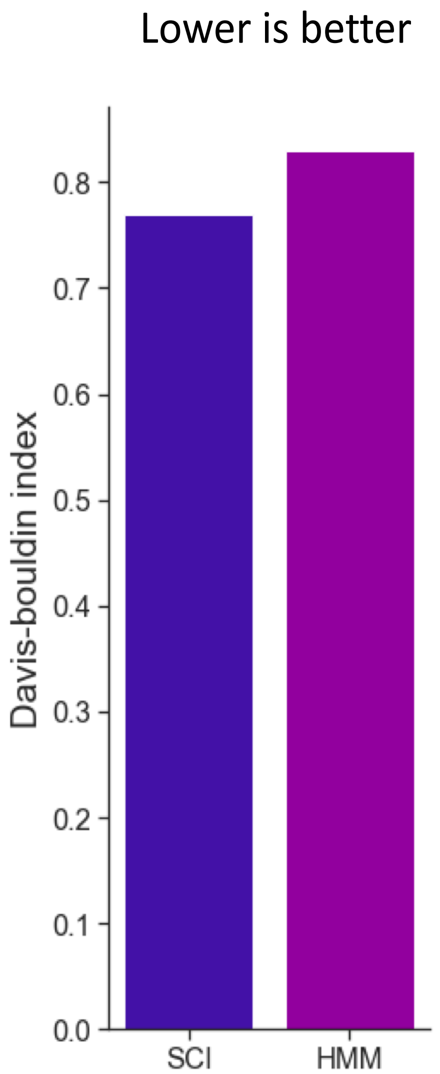

Supplementary Figure 4. Davies-Bouldin index for SCI and HMM sub-compartment predictions

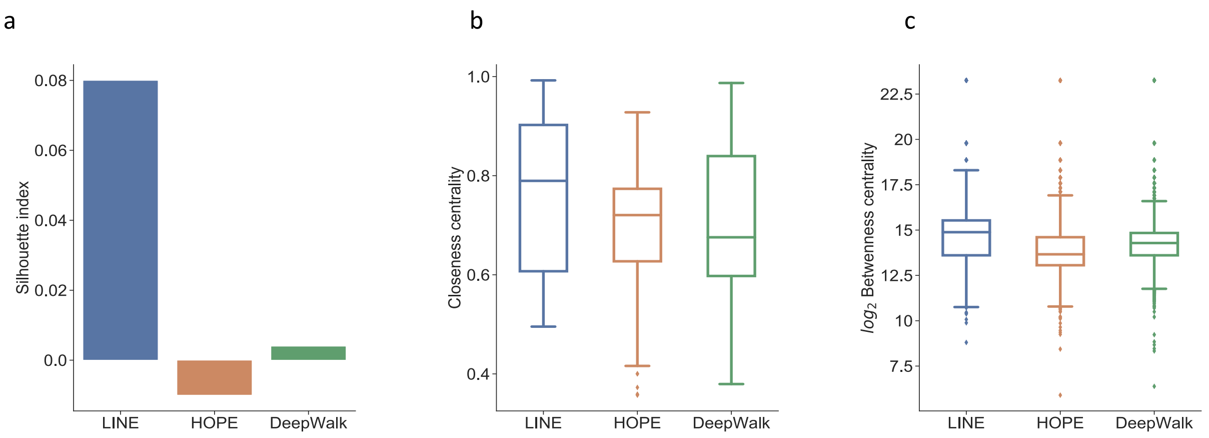

Supplementary Figure 5. Performance evaluation of alternative graph embedding algorithms (HOPE, DeepWalk). **a)** Bar plot for the Silhouette index. **b)** Boxplot for closeness centrality. **c)** Boxplot for betweenness centrality. For the boxplots, the top and bottom lines of each box represent the 75th and 25th percentiles of the samples, respectively. The line inside each box represents the median of the samples. The upper and lower lines above and below the boxes are the whiskers.

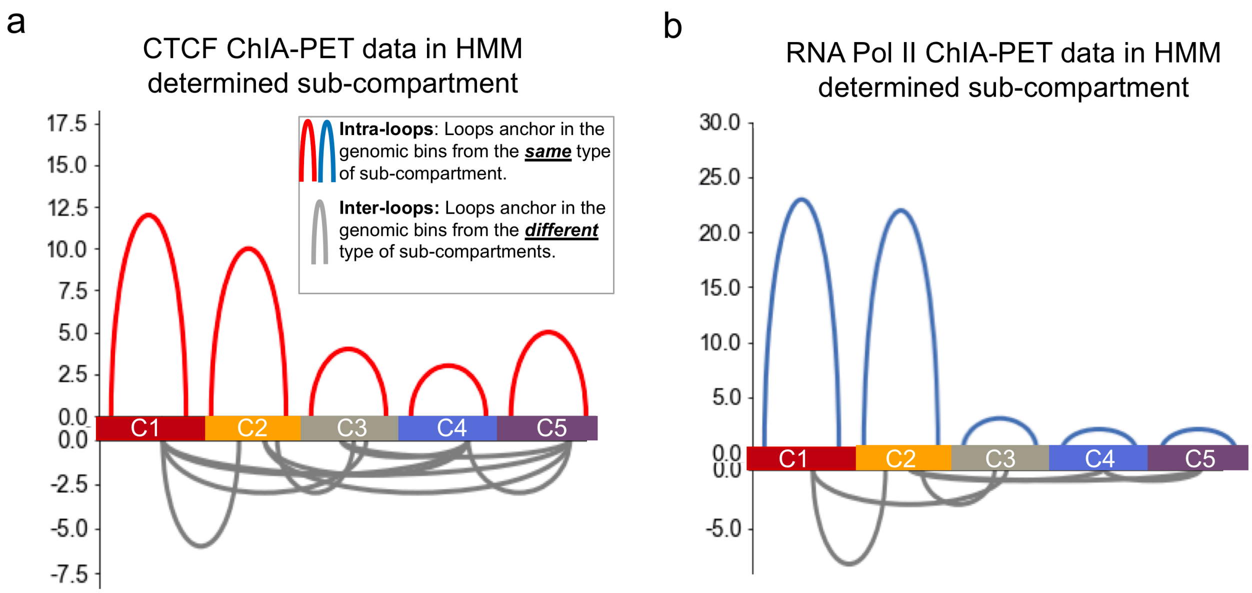

Supplementary Figure 6. CTCF and RNA Pol II ChIA-PET data validation for sub-compartments determined by HMM. a) CTCF ChIA-PET data validation. **b)** RNA Pol II ChIA-PET validation.

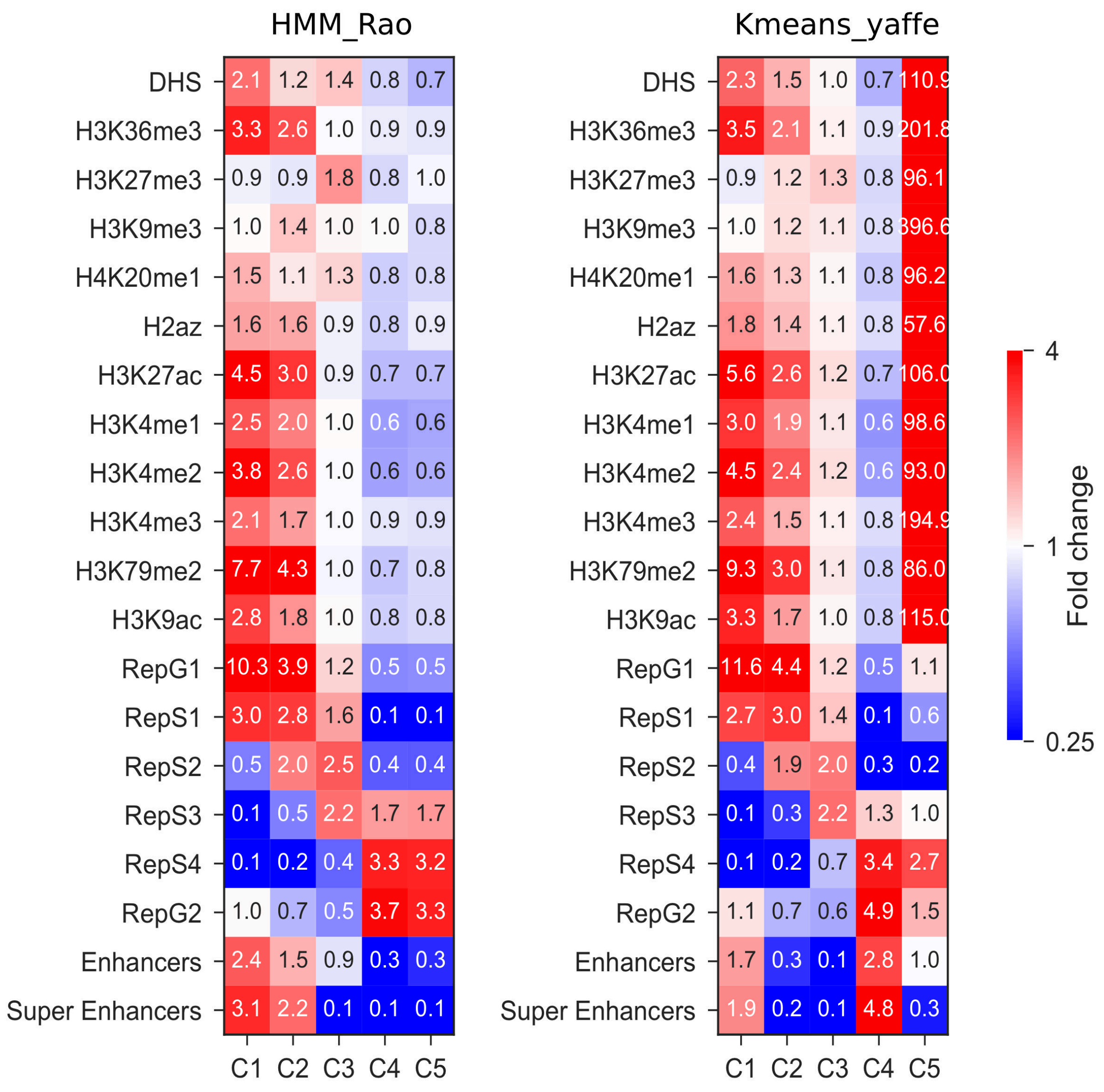

Supplemental Figure 7. Heatmap of enrichment of epigenomic marks in sub-compartments predicted by HMM_Rao and Kmeans_Yaffe.

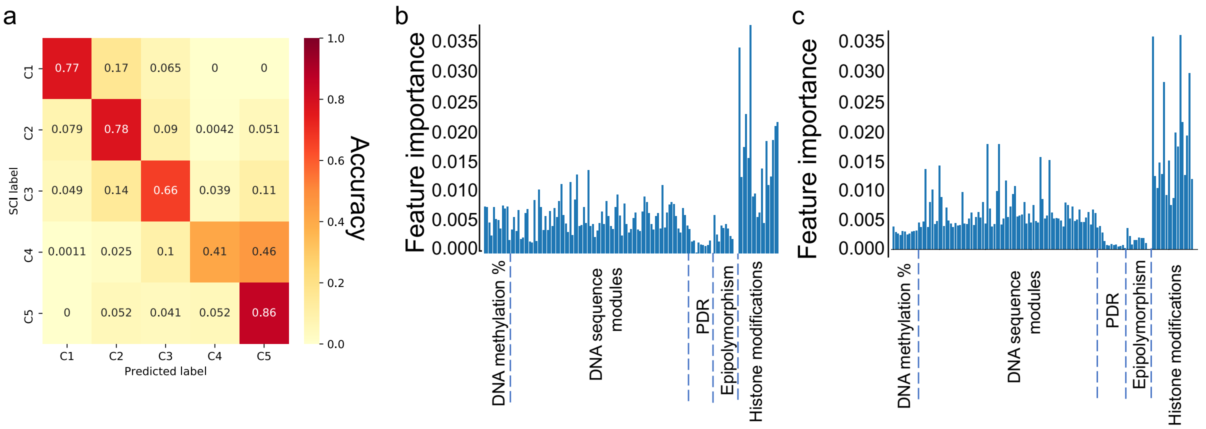

Supplementary Figure 8. Classification results for epigenome-based classifiers. **a)** confusion matrix of the deep neural network model prediction. **b)** Feature importance evaluated using XGBoost. c) Feature importance evaluated using the random forest.

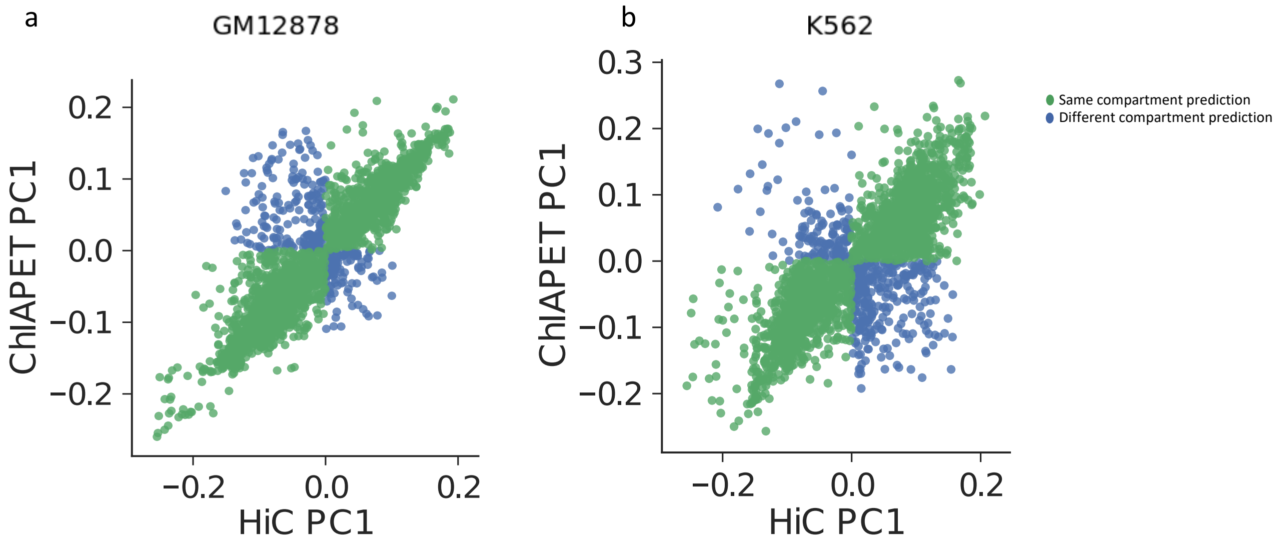

Supplementary Figure 9. Scatter plot of similarity between first Eigenvectors used to predict A/B compartments from Hi-C and ChIA-PET genomic bins.

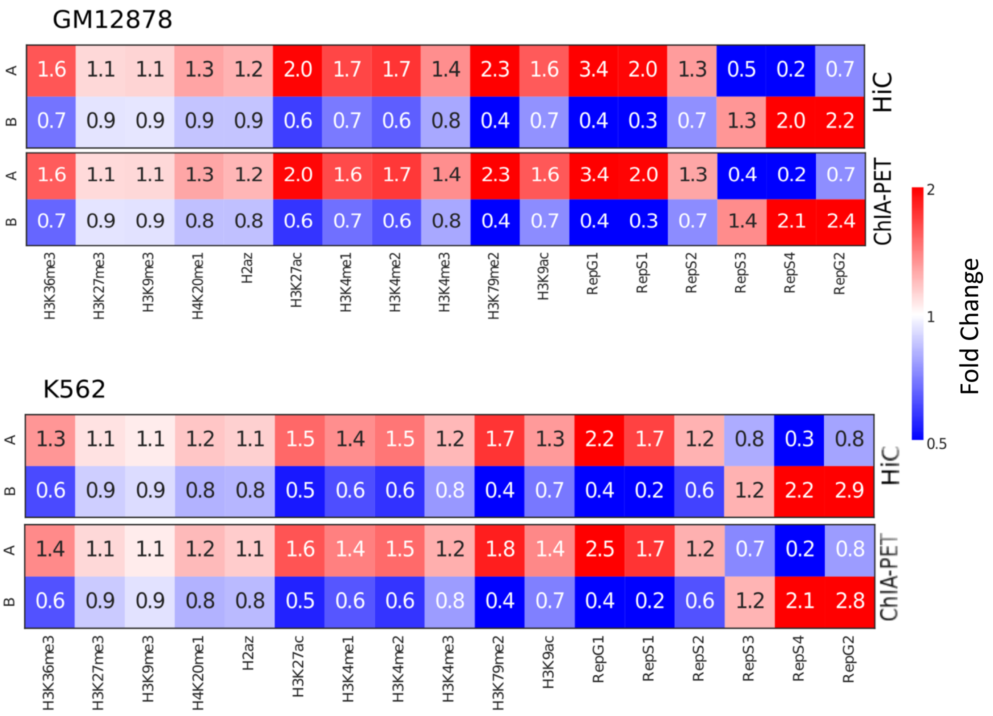

Supplementary Figure 10. A heatmap of enrichment patterns between Hi-C and ChIA-PET compartments using several epigenomic histone marks identified from ChIP-seq data and replication timing data (Repli-seq).

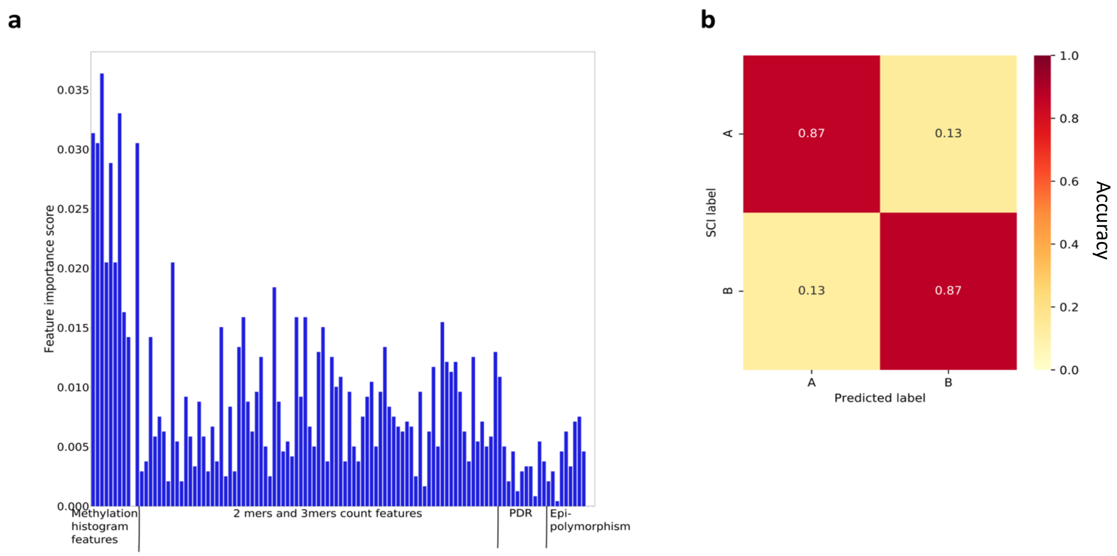

Supplementary Figure 11. Performance assessment of compartment predictor using input features, including DNA methylation and DNA sequence.

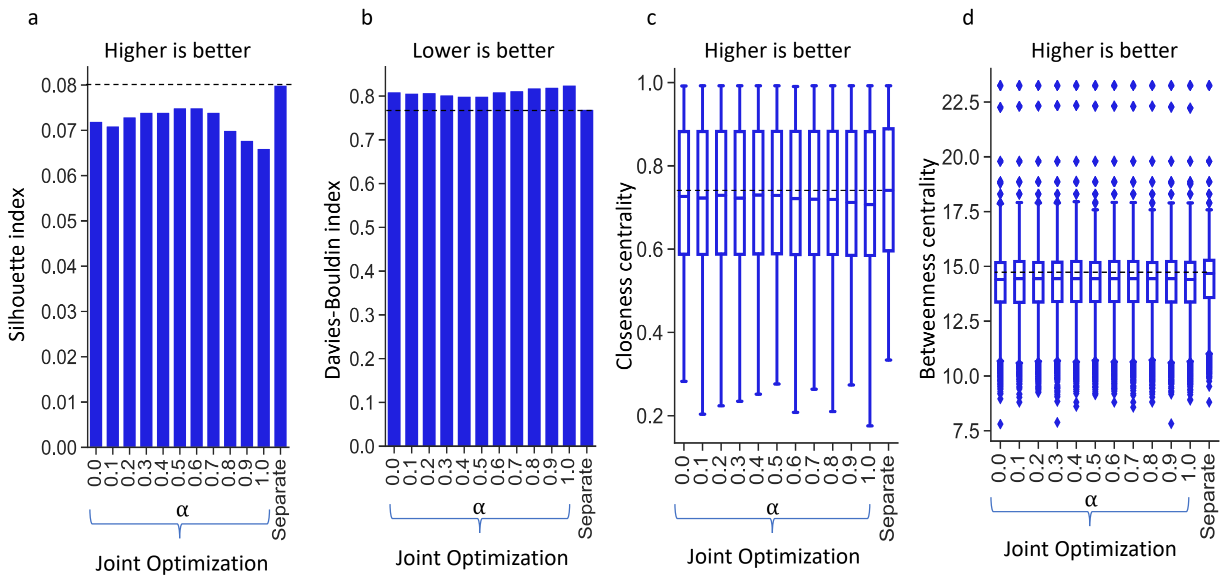

Supplementary Figure 12. Comparison between joint and separate optimization clustering performances using different metrics including a) Silhouette index, b) Davies-Bouldin index, c) closeness centrality, and d) betweenness centrality.

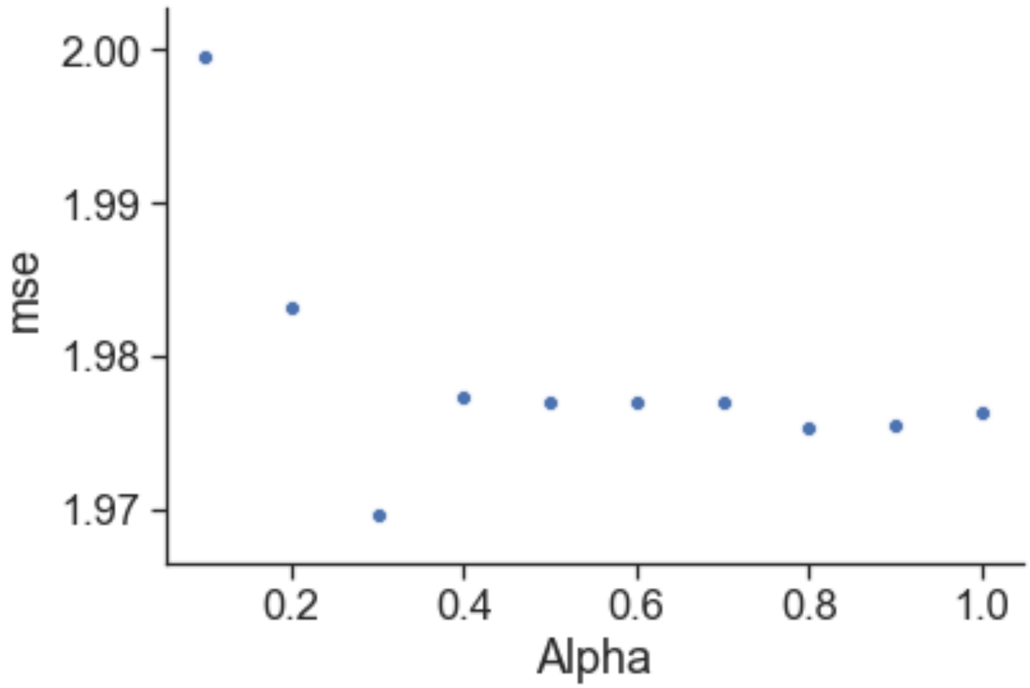

Supplementary Figure 13: Scatter plot shows different mean square errors (mse) when changing the elastic-net mixing parameter (alpha).

Supplementary Table 1. Transcription factors enriched in each sub-compartment as identified by elastic-net.

Sub-compartment	Enriched TFs
C1	YY1, ELK1, ELF5, ARNT, MYC, PROX1, ZBTB7B, GMEB2, SPIB, NFYA, E2F2, BRCA1, HSF4, ZNF238, E2F5
C2	YY1, ETV6, ELK1, ZBTB7B, E2F2, SNAI2, GMEB2, BATF, HOXA3, MTF1, EP300, AHR, ZHX1, NFYB, NR4A1, MYBL1, DRGX
C3	YY1, GMEB2, E2F5, ELK1, ARNT, ETV3, ELF4, ELK3, HNF4A, HSF4, YY2, ATF2, ETV, ERG, NR1I2, ZNF784, ETV5, KDM2B, MYBL1, HIF1A
C4	ETV5, ELF4, ETV1, TLX2, GATA4, STAT1, ATF1, PAX1, NKX2.1, SMAD3, HOXA7, RUNX2, HES7, SOX8, CBFB, NR4A2, ELK1
C5	YY1, ELK1, ELF4, NFYA, RFX1, YY2, CREB1, ZNF784, SPIC, NFYB, ELF3, ISL2

Supplementary Table 2. Accuracy comparison between sub-compartment prediction based on ChIP-seq data and whole genome bisulfite sequencing (SCI)

**Method**	**Test Accuracy on <u>even-numbered</u> chromosomes**
DNN model (using histone modification, DNA methylation, replication timing, and DNA sequence)	68%
DNN model (using methylation and DNA sequence)	61%
Di Pierro et.al[13] (84 ChIP-seq data sets)	66 %
Di Pierro et.al[13] (11 ChIP-seq data sets – histone modifications only)	54%
